# Development of an immunodiagnostic assay for the detection of *Sugarcane mosaic virus*

**DOI:** 10.55730/1300-0152.2714

**Published:** 2024-08-27

**Authors:** Hafiz Muhammad KHALID, Najam us Sahar Sadaf ZAIDI, Naeem RASHID, Muhammad TAHIR

**Affiliations:** 1 Department of Agricultural Sciences and Technology, Atta-ur-Rahman School of Applied Biosciences, National University of Sciences and Technology, Islamabad, Pakistan; 2 Department of Biological and Health Sciences, Pak-Austria Fachhochschule Institute of Applied Sciences and Technology, Haripur, Pakistan; 3 School of Biological Sciences (SBS), University of the Punjab, Lahore, Pakistan

**Keywords:** *Sugarcane mosaic virus*, polyclonal antibody, RT-PCR, immunodiagnostic, sugarcane

## Abstract

**Background/aim:**

*Sugarcane mosaic virus* (SCMV; genus *Potyvirus* and family *Potyviridae*), poses a significant threat to global sugarcane cultivars, including those in Pakistan. The aim of this study was to develop a rapid and effective diagnostic tool for detection of SCMV, enabling timely implementation of control measures to mitigate potential yield losses.

**Materials and methods:**

The study focused on the in silico analysis, physicochemical properties, immunogenicity, and subcellular localization of the SCMV coat protein (CP). The SCMV CP gene was synthesized, cloned, and expressed in *Escherichia coli*. The recombinant fusion CP (rFCP-SCMV) was purified and used to generate polyclonal antibodies (pABs) in mice. The immunogenicity of the antibodies was evaluated through indirect ELISA and RT-PCR.

**Results:**

Epitope prediction using tools like the OptimumAntigen design tool from GenScript, BepiPred, and IEDB identified key B-cell epitopes on the SCMV CP, enhancing the specificity of the antibodies. Structural modeling with SWISS-MODEL and PyMOL provided insights into the 3D structures of viral proteins and their epitopes, aiding in the design of high-affinity antibodies. Molecular docking studies simulated the interaction between antibodies and viral epitopes, enabling the selection of optimal antibody candidates. The synthesized recombinant fusion CP (rFCP-SCMV) was used to produce pAbs in mice. These antibodies exhibited high sensitivity, detecting as low as 100 pg of SCMV protein in indirect ELISA. They also effectively identified SCMV in infected sugarcane field samples, confirmed by RT-PCR. The antibodies maintained high specificity and sensitivity even at a 1:10,000 dilution, proving their efficacy in recognizing both the recombinant protein and virus particles in plant sap.

**Conclusion:**

The study reveals a rapid, effective immunodiagnostic technique for detecting SCMV in sugarcane cultivars, offering an accurate alternative to conventional virology methods, reducing contamination risk, and providing a valuable tool for mitigating yield losses.

## 1. Introduction

Sugarcane (*Saccharum* spp.) is among the most important economic and commercial crops in tropical and subtropical regions of the world (Furtado Júnior et al., 2020). The mosaic caused by the *Sugarcane mosaic virus* (SCMV) is one of the most devastating diseases of sugarcane, not only significantly affecting sugarcane productivity (Akbar et al., 2017a; Darsono et al., 2018; Lu et al., 2021) but also interfering with the production of maize, sorghum, *Canna*, and other gramineous species worldwide (Viswanathan et al., 2018; He et al., 2020; Xu et al., 2021; Muhammad et al., 2022). Incidences of SCMV have been reported in at least 70 countries (Akbar et al., 2020; Moury and Desbiez, 2020). According to a recent study, SCMV has spread throughout sugarcane plantations in Pakistan, with a 7.01% incidence rate (Nisa et al., 2022) and a 20% decline in sugar production (Haider et al., 2011; Akbar et al., 2017b).

The SCMV genomic is around 10 kb in size and is a single-stranded positive sense RNA (Wylie et al., 2017; Yahaya et al., 2019; He et al., 2020). The polyprotein produced by SCMV was cleaved, co- and posttranslationally, into its component gene products, including the coat protein (CP) gene, by viral coded proteases (Hema et al., 2001; Zhu et al., 2014; Tang et al., 2016). Since the CP is predominantly conserved among SCMV isolates, serological methods can be used to detect the virus (Čeřovská et al., 2003).

Plant viruses have been widely detected using numerous immunodiagnostic techniques as well as molecular techniques (Hema et al., 2001; Fu et al., 2015; Viswanathan et al., 2018; Lu et al., 2021). Generally, molecular approaches for viral detection are not appropriate for large-scale sample indexing as routine tests since they are more expensive, and the technique is more complicated than serology-based detection (Thorat et al., 2015).

Traditionally, a polyclonal antibody is developed using a pure virion from infected plant material for immunodiagnostics (Hourani and Abou-Jawdah, 2003). However, the traditional approach to producing polyclonal antibodies has two drawbacks: the minimum viral concentration in the infected plant tissue and the purity of the virion. The process of propagating a virus in an appropriate host and purifying it is laborious (Hull, 2013). Moreover, certain viruses, such as potyvirus and phloem-limited luteoviruses, are particularly difficult since they have low viral concentrations.

In the present study, we utilized in silico methods to enhance the development of a diagnostic tool for SCMV detection. Computational analyses, including examination of the physicochemical properties, immunogenicity, and subcellular localization of the SCMV CP, were used to predict the protein’s behavior and optimize its antigenic properties (Mortazavi et al., 2024). These in silico approaches provide valuable insights that guide the design of recombinant proteins and improve the sensitivity and specificity of immunodiagnostic assays. By integrating in silico predictions with experimental validation, we aim to develop a rapid and reliable diagnostic tool for SCMV. This approach not only enhances the accuracy of SCMV detection but also reduces the time and resources required for diagnostic procedures, offering a significant advancement over traditional methods (Oli et al., 2020).

Recombinant CP-based antibodies have been utilized extensively in immunodiagnostics to address the problem. Recombinant CPs have been used in several investigations as antigens to produce polyclonal antibodies, including *Cucumber mosaic virus* (Khan et al., 2011), Citrus *psoriasis virus* (Salem et al., 2018), *Sugarcane mosaic virus* (Darsono et al., 2018), and *Onion yellow dwarf virus* (Kumar et al., 2021). According to a recent report (Hoffmann et al., 2022), CP-based antisera can detect viruses in symptomless primary and secondary hosts. Although the application of CP-based antisera for virus detection in symptomless plants may not constitute a novel approach, it is important to take into account that this method has a rich history in scientific research. For decades, researchers have relied on antisera specifically designed to target the CP of purified virus particles. This long-standing tradition highlights the enduring effectiveness and practicality of CP-based antisera in the field of virus detection; thus, serological detection is an easy, quick, and affordable technique suitable for virus identification. Therefore, crop damage can be reduced, and customized treatments can be used to tackle the pathogens.

In the present study, the CP gene of an SCMV isolate infecting sugarcane in Pakistan was overexpressed in a bacterial expression system (*E. coli*) and the recombinant fusion CP was purified and mobilized in mice for the development of polyclonal antibodies. In addition, the optimization, validation, and standardization of ELISA immunodiagnostics were reported herein.

## 2. Materials and methods

### 2.1. Phylogenetic and physicochemical analysis

The CP gene sequence of the SCMV isolate (accession number AJ278405) was obtained from GenBank^1^ and subjected to alignments by MUSCLE software, built into MEGA X v10.1.8 (Kumar et al., 2018) and phylogenetic analysis was performed by the maximum-likelihood (ML) method, using the codon-based alignments with 1000 bootstrap replicates. The physicochemical characteristics were meticulously computed using the widely recognized ExPASy ProtParam online tool^2^ (Gasteiger et al., 2005). A 3D structural model was generated using I-TASSAR^3^ (Zhou et al., 2022) and SWISS-MODEL^4^ (Waterhouse et al., 2018), respectively. PyMOL-2.5.7^5^ was used to illustrate the structure produced by the I-TASSER server’s PDB data (Delano, 2002) (Supplementary Figure S1). The presence of signal peptides and cellular localization was predicted using online web servers^6^ like SOSUI series system^7^ (Mitaku et al., 2002) and PSORTb version 3.0.3^8^ (Yu et al., 2010).

### 2.2. Immunogenic analysis

Immunogenic analysis was performed using the immune epitope database (IEDB)^9^ (Vita et al., 2019) and the Optimum Antigen™ design tool (GenScript, Piscataway, NJ, USA)^10^ and their potential B-cell epitopes were predicted with a default threshold of 0.1512 by BepiPred-3.0 Linear Epitope Prediction tool^11^ (Supplementary Figure S2) (Clifford et al., 2022).

### 2.3. Transformation and subcloning expression cassette

The final expression cassette (SCMVCpaj405) was commercially synthesized from Macrogen Inc. Korea in pUC57 and subsequently cloned in pET28 (a+) vector using restriction endonuclease sites *Nde*I (Cat #ER0581) and *Hind*III (Cat #R008S) to generate the 6×His-tagged SCMVCpaj405- pET28 (a+) plasmid, which was then used to transform DH5 α (*E. coli*) competent cells. Positive transformants were screened using restriction digestion and DNA sequencing before being used to transform *E. coli BL21* CodonPlus competent cells for gene expression.

### 2.4. Expression optimization and purification of fusion protein

The expression of the 6×His-SCMVCpaj405 fusion protein was induced by adding the synthetic inducer, IPTG, at concentrations of 0.1, 0.5, and 1 mM for 3 and 6 h at 37 °C. The bacterial cultures were centrifuged for 15 min at 4000 rpm and 4 °C. The resultant pellets were suspended in 50 mM Tris-HCl pH 8.0 buffer and sonicated for 30 min on ice with a pulse of 20-s on, 40-s off cycle to disturb them. The total protein extracts were separated into fractions (soluble and insoluble) by centrifugation at 12,000 rpm for 20 min at 4 °C prior to the large-scale production of recombinant protein, which was induced for 6 h at a concentration of 0.5 mM IPTG. The purification was carried out under native conditions (as a soluble protein) using Ni-NTA (resin Bio-Rad Laboratories, Inc. California, USA) affinity chromatography. To remove the imidazole, the purified recombinant protein fractions were mixed and dialyzed against a phosphate buffer saline (pH 8.0) overnight at 4 °C. The protein’s purity was verified on 12% SDS-PAGE. The Bradford assay (Bradford, 1976) was used to determine the protein concentration by using bovine serum albumin (BSA; Merck, Germany) as a standard protein. This recombinant protein was then utilized as an antigen to develop mouse-based polyclonal antibodies in the following phase.

### 2.5. Production of mouse polyclonal antibodies and serum collection

The animals were housed in a controlled environment (temperature, light, and 12-h light/dark cycle) at the School of Biological Sciences (SBS), University of the Punjab, Lahore, Pakistan, where they always had access to food and water. Mouse polyclonal antisera to fusion protein were raised in albino female mice (Swiss Webster white strain, 4–6 weeks old) upon the approval by the Ethics Committee of SBS according to the protocol outlined by Salem et al. (2018). The mice were given an initial dose of 100 g of recombinant CP with the same volume of Freund’s complete adjuvant (Santa Cruz Biotechnology, Dallas, TX, USA), followed by four weekly intraperitoneal booster injections of 200 g without removing the 6×His-tag with incomplete adjuvants (Santa Cruz Biotechnology). The mice were given deep anesthesia with ketamine hydrochloride (50 mg/kg of animal body weight) and xylazine (5 mg/kg of animal body weight) through intraperitoneal administration prior to blood collection through the cardiac puncture (Sun et al., 2021). The blood was centrifuged at 4000 rpm after being incubated for 1 h at 37 °C and antisera were stored at −20 °C for further analysis.

### 2.6. Optimization of ELISA

#### 2.6.1. Optimization of raised antisera and antigen

According to the standard protocol, three independent experiments were performed for the preparation and characterization of raised antibodies in mice. Raised antisera and antigen coating conc was optimized by serial dilution method (i.e. 50X, 100X, 1000X, 10,000X) as outlined by Darsono et al. (2018) with modification of a dilution factor extended from 50X to 10,000X to accommodate the specific requirements of our study. The antigen (200 μL; 100 ng/well) was prepared in borate buffer (50 mM KCl borate, pH 8.0) and used to coat the wells of a microtiter plate. The next day, the coating solution was removed and washed three times with 1X PBST buffer with continuous shaking for 5 min. The vacant binding sites were saturated with a blocking reagent to reduce nonspecific binding to the surface, and 200 μL of blocking buffer (5% (W/V) skim milk in TBS-T) was added to each well followed by incubation at 37 °C for 2 h. After incubation, the wells were cleaned as before and dried. To detect the immunoreactivity the anti-mouse HRP conjugated IgG antibody produced in rabbits (A9171 Sigma, Merck KGaA, Darmstadt, Germany) was diluted (50,000X) in blocking buffer and added to each well (200 μL/well). After incubation at 37 °C for 1 h, the wells were washed three times with 1X PBST buffer with continuous shaking for 5 min (200 μL/well) and dried. The plate was incubated at RT for 15 min for blue color development. The reaction was stopped by the addition of 2 M H_2_SO_4_ (100 μL/well). To analyze the antibody titer, the differential OD450/630 absorbance was measured at 405 nm using a microplate reader (HUMAREADER plus, human GMBH). All the mice’s serum samples were replicated twice for their immunoreactivity, the values were recorded, and averages were calculated. The standard curve was plotted for different concentrations of antigen, i.e. 10 pg, 50 pg, 100 pg, 200 pg, 500 pg, 1 ng, 5 ng, 50 ng, 100 ng, 200 ng, 500 ng, 1 μg, and 2 μg were used in the optimization process with immunized mice and negative controls. The rest of the ELISA procedure followed the same methodology as described before.

### 2.7. Indirect ELISA

In the indirect ELISA, the sensitivity of the raised antisera was evaluated by serial dilution (Darsono et al., 2018). The effectiveness and specificity of the raised antiserum in detecting the SCMV in various test samples were evaluated using targeted purified fusion protein, bacterial cell extracts (both recombinant and nonrecombinant plasmids), and total proteins extracted from healthy and SCMV-infected sugarcane plants (Salem et al., 2018).

### 2.8. RT-PCR

Using TRI REAGENT^®^ (Cat. #TR118), total RNA was meticulously extracted from 0.1 g of plant leaf tissues sourced from the field, encompassing both healthy samples and those displaying characteristic mosaic symptoms suggestive of mosaic disease for subsequent RT-PCR analysis. For the confirmation of the SCMV-infected sugarcane sample, the CP gene-specific primers (SNd-Cp-F CATATGGCTG-GAACAGTCGATGC and SHd-Cp-R AAGCTTCTAGTGGTGCTGCTGCAC) were designed using Primer3Plus and used in QIAGEN OneStep RT-PCR analysis, according to the manufacturers’ instructions.

### 2.9. Statistical Analysis

Before the statistical analysis, the data were checked for whether they were normally distributed or not. Then with the help of one-way analysis of variance (ANOVA), the Tukey post-hoc test, and GraphPad Prism software 8.0 (USA), statistical analysis and inferences were carried out. According to the Tukey post-hoc test using the least significant difference test, comparisons between treatments were deemed statistically significant at p < 0.05. All experiments were replicated twice. Data from three independent experiments are presented as mean and standard error.

## 3. Results

### 3.1. Physicochemical and immunogenic analysis

**Figure 1:**
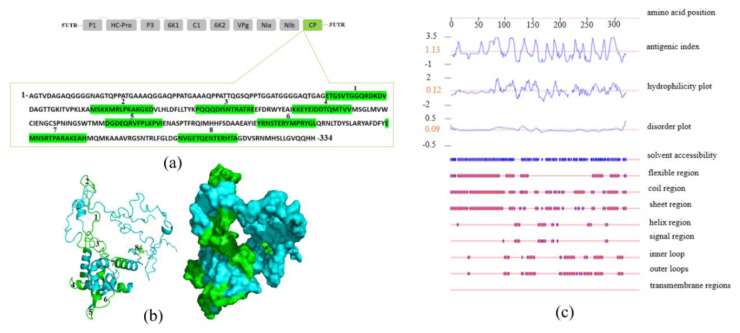
Genome organization and amino acid sequence of the SCMVCpaj405 protein (a), amino acid sequence and schematic structures indicate the antigenic determinant regions located in SCMVCpaj405 protein (highlighted in green) (b), and in silico analysis of the immunogenetic determinant property of the SCMVCpaj405 protein (c).

The physicochemical characteristics of native and fused CP-SCMV protein sequences were compared using ExPASy ProtParam (Table 1). The nucleotide sequence translation to amino acids showed that both the native and 6×His tag CP genes code for 314 and 321 aa proteins, respectively. The computational prediction of isoelectric point (pI) and molecular weight of the proteins showed that they have pI values approximately the same, with native CP having a pI of 6.80 and the 6×His tag CP having a pI of 7.00 with theoretical protein mass of 33.8 kDa (native CP) and 34.7 kDa (6×His tag CP). The predicted protein size for rCP is around 34.7 kDa since the pET 28a (+) vector adds a few amino acid linkers and a 6×His tag to the protein’s N-terminus, adding roughly ~1 kDa (Koh et al., 2020). Two different tools (PSORTb and SOSUI) were used to determine the subcellular localization of native and fused proteins in *E. coli*. The PSORTb result revealed an unknown subcellular localization for both proteins; however, the SOSUI analysis showed a periplasmic subcellular localization for both proteins. The soluble proteins were predicted by the tools SOSUI signal and Signal P 5.0 without a signal peptide (Supplementary Figure S3, Supplementary Tables S1 and S2). Furthermore, the antigenic determinants of CP-SCMV were located in eight distinct stretches: ETGSVTGGQRDKDV, MSKKMRLPKAKGKD, PQQQDISNTRATRE, KKEYEIDDTQMTVV, DGDEQRVFPLKPVI, YRNSTERYMPRYGL, EMNSRTPARA-KEAH, and NVGETQENTERHTA, which were present at the upper surface of the 3D structure (Figures 1a–1c) (Supplementary Table S3).

### 3.2. Production and purification of 6×His-SCMVCpaj405 fusion protein

**Figure 2:**
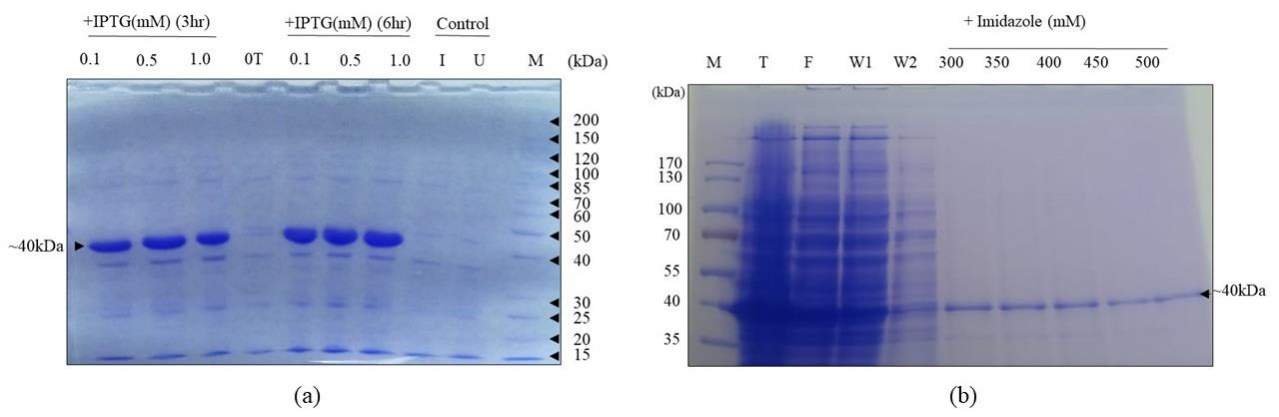
Overexpression and purification analysis of SCMVCpaj405 protein. (a) Effect of inducer concentration (IPTG) on the SCMVCpaj405 expression level; (b) purification of the SCMVCpaj405 protein using Ni-NTA affinity in different imidazole concentrations. 0T is the protein fraction collected before the IPTG induction. The pET28 (a+) without the insert was used as control (I = induced; U = uninduced) SCMVCpaj405 protein soluble fraction (T), flow-through (F), washing (W, W1), and unstained protein marker (M) are shown.

A 40 kDa overexpressed protein band was observed in BL21 DE3 culture in setups transformed with the 6×His-SCMVCpaj405-pET-28a (+) vector (Supplementary Figures S4 and S5) and induced by IPTG (0.1, 0.5, 1.0 mM, 3 h and 6 h) at 37 °C, as shown in Figure 2a. When examining the intensity of the 40 kDa band size for 3 h and 6 h of incubation, there was an increase in band size. Protein (Supplementary Figure S6) was purified under native conditions. SDS-PAGE analysis of elution fractions (450 and 500 mM of imidazole) under native conditions using the Ni-NTA column confirmed that the eluted protein was SCMVCpaj405 tagged with 6×His (Figure 2b). Protein elutions were mixed, and dialysis was used to eliminate extra imidazole. Using BSA as the standard, the resultant solution was subjected to the Bradford test to determine the eluted protein concentration. According to the findings, 2 mg of recombinant SCMVCpaj405 protein was produced per 200 mL of BL21 (DE3) culture (Bradford, 1976).

### 3.3. Development and evaluation of polyclonal antibodies

**Figure 3:**
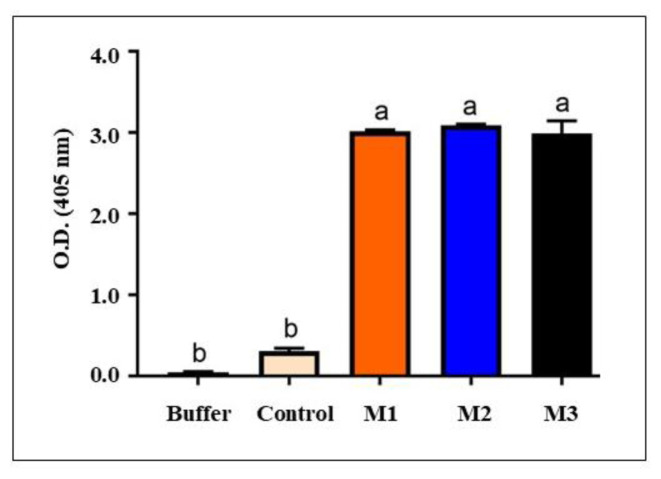
Serological analysis to evaluate the antibody production in immunized mice (C1, C2, C3) versus unimmunized mice (control).

**Figure 4:**
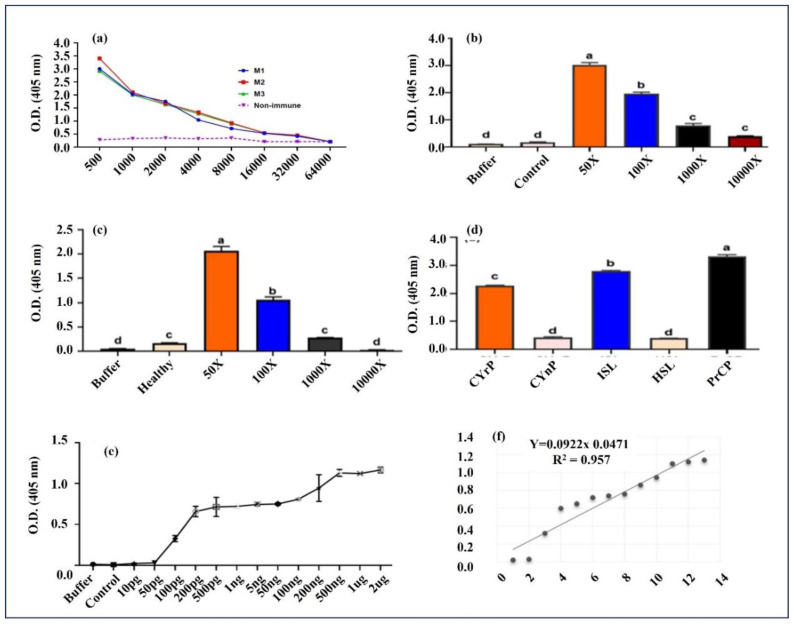
Optimization of indirect ELISA for SCMV detection. (a) Polyclonal antibody titer assessment in the immunized mice (M1, M2, M3) compared to the control mouse. (b) Primary antibody coating concentration optimization using serial dilution. (c) Sensitivity analysis of raised antibodies using varying dilutions of protein lysates from SCMV-infected sugarcane plants. (d) Evaluation of antiserum effectiveness and specificity against various test samples. (e) Standard curve generated for antigen optimization through serial dilution. (f) Linear regression analysis demonstrating a working range of 100 pg/mL to 500 ng/mL (R^2^ = 0.957). Letter labels (a, b, c, d, etc.) indicate statistically significant differences between buffer, control, and immunized mice. M1, M2, and M3 represent mice immunized with the antigen and Freund’s adjuvant, while the control mouse received (1X PBS) buffer and Freund’s adjuvant.

Initially, all three immunized and nonimmunized (control) animals’ antisera were tested for antibody production using indirect ELISA. The ELISA results for the immunized mice were positive and virtually comparable, in contrast with the nonimmunized mice, which showed negative results (Figure 3). To establish the optimal coating concentration for the raised primary pAb antibody, serial dilution was used with a fixed amount of pure SCMVCpaj405 fusion protein antigen (100 ng/well) (Figure 4a). Antisera targeting the SCMVCpaj405 fusion protein effectively detected the antigen across a wide dilution range, from 50X to 10,000X. The mean absorbance at 405 nm of the SCMVCpaj405 fusion protein antigen exhibited a gradual decrease, ranging from 3.094 (50X) to 1.719 (10,000X). Notably, the absorbance values of the buffer (0.1135) and healthy control (0.183) were consistently lower than that of the antigen (Figure 4b). The purified SCMVCpaj405 fusion protein antigen remained readily identifiable up to a 10,000X dilution, where the mean absorbance value of 1.719 surpassed the established threshold value (TV) of 0.312. A sample was deemed significant if its mean absorbance value exceeded the TV.

The sensitivity of the antisera was further evaluated by using indirect ELISA analysis at dilutions ranging from 50X to 10,000X to crudely extracted proteins from both healthy and infected sugarcane leaves. The crude extracts’ dilution steadily dropped, from 2.0 (50X) to 0.028 (10,000X) dilution at A405 nm. The raised antibody generated the strongest signal to identify virus particles in plant sap that had been diluted 1:50 and 1:100 (Figure 4c), with letters (a, b, c, d, etc.) showing statistically significant differences between variables.

Indirect ELISA was also used to evaluate the effectiveness and specificity of the raised antiserum to various test samples. The raised antibodies reacted positively against purified fusion protein, total cell lysate from bacterial cells transformed with recombinant plasmid (Figure 4d), and total extracted proteins from SCMV-infected sugarcane plant in the indirect ELISA. Nevertheless, no signal was detected against the nonrecombinant plasmid extract or in the healthy plant total extract (Table 2).

### 3.4. The standard curve

**Figure 5:**
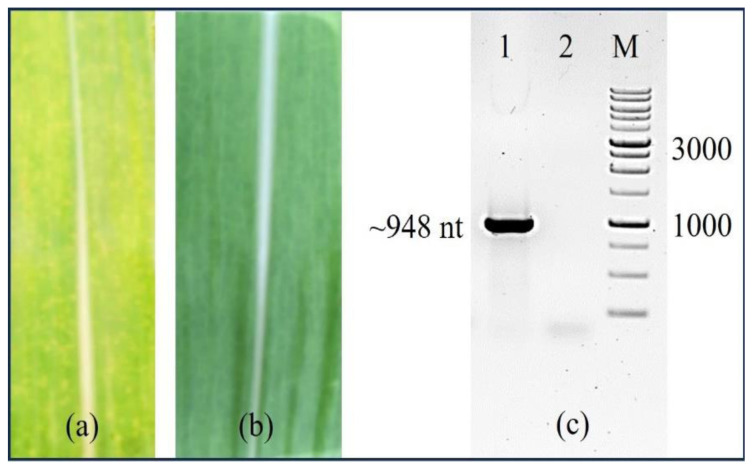
Detection of SCMV infection in sugarcane leaves using RT-PCR. Visual comparison of (a) symptomatic and) nonsymptomatic sugarcane leaves. (c) RT-PCR amplification of the SCMVCpaj405 protein gene using symptomatic (1) and healthy (2) leaf samples, with M representing a 1 kb DNA ladder.

To quantitatively measure SCMV in plant tissues using indirect ELISA, a standard curve was established by serially diluting SCMVCpaj405 fusion CP (10 pg to 2 μg). The resulting linear regression between SCMVCpaj405 fusion CP concentration and OD450 absorbance was described by the equation y = 0.0922x + 0.0471 with a correlation coefficient (R^2^) of 0.957. This analysis indicated a minimum detection limit of approximately 100 pg/mL for SCMVCpaj405 fusion CP. Furthermore, the linear regression model demonstrated a working range of 100 pg/mL to 500 ng/mL (Figures 4e and 4f). Finally, PCR-based confirmation was performed with SCMVCpaj405-specific primers SNd-Cp-F and SHd-Cp-R using the same healthy and SCMV-infected sugarcane field-grown plant used in the ELISA. Total extracted RNA was used as a template to create 1st strand cDNA by reverse transcription, and one-step RT-PCR successfully amplified the full length SCMV CP gene from infected sugarcane field-grown plant but not in a healthy one, which confirmed the presence of SCMV (Figure 5).

## 4. Discussion

SCMV significantly impacts sugarcane yield and quality, posing a challenge for the production of healthy, virus-free planting material, given that sugarcane is predominantly propagated through vegetative means. A high-throughput diagnostic method is crucial for ensuring the quality of planting material. Essential to such diagnostics are high-quality antisera, which can be produced by generating polyclonal antibodies against recombinant viral proteins.

In the present study, we successfully subcloned the CP gene of SCMV (AJ278405.1) and expressed it in *E. coli* BL21 (DE3) cells. CP is frequently utilized as an antigen for polyclonal antibody production because it is highly conserved across different virus strains. This conservation allows broad-spectrum detection of SCMV, whether the virus is in an active replication phase or in an inactive state (Xie et al., 2007; Li et al., 2015; Yang et al., 2019).

The pET28a (+) bacterial expression vector, equipped with a T7 promoter for protein expression in *E. coli* and an N-terminal 6×His tag for purification and detection, was chosen due to the anticipated nonimmunogenicity of the 6×His tag (Mutasa-Gottgens et al., 2000; Kumari et al., 2001; Gulati-Sakhuja et al., 2009; Le et al., 2022). As expected, the 6×His tag facilitated efficient affinity purification of the recombinant protein without compromising the diagnostic potential of the polyclonal antiserum. Induction of transformed *E. coli* BL21 (DE3) cells with 0.5 mM IPTG successfully yielded recombinant SCMV CP, with protein production increasing proportionally to the postinduction incubation time (Figure 2a).

Purification of the recombinant SCMV CP under native conditions revealed its predominant localization in the soluble fraction of *E. coli* whole-cell protein extract. The purified protein exhibited a molecular size of approximately 40 kDa, which was larger than the predicted 34.7 kDa. This observation aligns with previous findings by Jensen et al. (1986) that SCMV-induced CP size can vary among isolates in plant tissues, ranging from 34.4 to 39.7 kDa (Figure 2b). Furthermore, it is consistent with studies on recombinant CPs from other plant viruses such as tomato spotted wilt tospovirus (Vaira et al., 1996), apple stem grooving virus (Nickel et al., 2004), and Citrus psorosis virus (Salem et al., 2018). The recombinant fusion protein was observed to be predominantly in the soluble phase.

Evaluation of the antibody titer involved assessing the raw antiserum’s ability to recognize the target antigen in SCMV-infected sugarcane samples. The results indicated that the mouse anti-SCMV CP antibodies could successfully react to SCMV-infected sugarcane samples up to a dilution of 1:10,000 of raw antiserum (Figure 4b). While cross-reactivity between antisera and their corresponding antigens is a potential concern, it is expected due to the frequent amino acid sequence and structural similarities among plant viral antigens within the same virus family, as documented in prior research (Hull, 2013; Koh et al., 2020).

The primary objective of immunodiagnosis in asymptomatic sugarcane plants is to determine the presence or absence of viral infections, regardless of the specific SCMV strain. Precise viral identification, a distinct goal, is better achieved through more specialized diagnostic techniques like PCR, monoclonal antibodies, or nanobodies (Llauger et al., 2021). The concordance of indirect ELISA and PCR findings to identify SCMV in sugarcane shows that the anti-SCMV CP is applicable for SCMV detection in field-collected samples.

The present study highlights the advantages of recombinant DNA technology over traditional viral purification methods, offering efficient bulk antigen synthesis for immunization. While native viral proteins from isolated virus particles maintain their original conformation, recombinant proteins can also be produced with similar structural integrity. The 6×His tag, shown to be beneficial in previous research, can be retained while optimizing protein expression conditions like incubation temperature and IPTG concentration to enhance solubility in *E. coli* (Raikhy et al., 2007; Koh et al., 2020).

Our findings and prior studies indicate that overexpressed proteins in *E. coli* often localize to the soluble phase. In silico predictions using SOSUI-GramN aligned with this observation, suggesting a periplasmic subcellular location for SCMV CP. Elfageih et al. (2020) reported that alkaline phosphatase facilitates the correct folding of proteins with disulfide bonds in the periplasm. Consequently, recombinant viral proteins expressed in *E. coli* can be reliably purified under native conditions, preserving their overall native conformation.

However, our work demonstrates that recombinant DNA technology is a productive and economical method of producing polyclonal antibodies against SCMV CP. In order to enable efficient disease control within sugarcane plantations, the generated antisera against SCMV CP may be effectively employed for the detection of SCMV in asymptomatic sugarcane plants. Furthermore, future research that makes use of the preliminary results from the present study can address the problem of specificity to further distinguish or discriminate between SCMV strains-infected sugarcane.

## 5. Conclusions

In the present study, the antibodies developed against the SCMV CP proved effective for detecting both the purified CP and the virus itself using ELISA. The availability of these antibodies enhances the ability to screen plant material in sanitation programs and supports research into the virus’s pathogenicity.

## Supplementary Data

Figure S1 3D model structures of SCMVCpaj405 protein constructed through I-TASSER (a) and SWISS-MODEL (b).

Figure S2 The graphical user interface for BepiPred-3.0 on Cp-SCMV protein. In this interface, the x and y axes are protein sequence positions and BepiPred-3.0 epitope scores. Residues with a higher score are more likely to be part of a B-cell epitope.

Figure S3 Graphical representation of signal peptide for SCMVCpaj405 protein by Signal-5.0 prediction.

**Table S1:** Signal peptide score summary of SCMVCpaj405 predicted by SignalP-5.0 for gram-negative bacteria.

# ID	Prediction	SP(Sec/SPI)	TAT(Tat/SPI)	LIPO(Sec/SPII)	OTHER	CS Position
Native SCMVCpaj405 protein	OTHER	0.296653	0.169714	0.054106	0.479527	OTHER
SCMVCpaj405 fusion protein	OTHER	0.218500	0.265910	0.023382	0.492208	OTHER

**Table S2:** Predicted subcellular localization site of SCMVCpaj405 native and fusion proteins by SOSUI_GramN_.

ID	seg.Length	Subcellular Localization site
Native SCMVCpaj405 protein	314a.a.	P (Periplasm)
SCMVCpaj405 fusion protein	334a.a.	P (Periplasm)

**Table S3:** Predicted antigenic epitopes of SCMVCpaj405 fusion protein by GenScript OptimumAntigen™ Design Tool.

No	Start	Antigenic Determinant	Length	Antigenicity/Surface/Hydrophilicity	Disordered Score	Synthesis	Mus_musculus blast
1	76	ETGSVTGGQRDKDVC	14	3.02/0.93/0.78	0.1466	Easy	49%
2	293	C NVGETQENTERHTA	14	2.78/0.86/0.78	0.1161	Easy	57%
3	105	MSKKMRLPKAKGKDC	14	2.61/0.71/0.96	0.1332	Easy	50%
4	130	C PQQQDISNTRATRE	14	1.84/0.86/0.67	0.1675	Easy	42%
5	259	CEMNSRTPARAKEAH	14	1.59/0.86/0.63	NONE	Easy	70%
6	228	CYRNSTE RYMPRYGL	14	1.41/0.50/0.34	NONE	Easy	50%
7	191	CDGDEQRVFPLKPVI	14	1.19/0.57/0.42	NONE	Normal	70%
8	153	CK KEYEIDDTQMTVV	14	1.19/0.57/0.47	0.1015	Normal	49%

Warn: peptide‐‐> PQQQDISNTRATRE:130 has no net charge, which may lead to dissolubility issues! Warn: peptide‐‐> YRNSTERYMPRYGL:228 consists of the candidate glycosylation sites!

**Note:**
1. An extra “C” (high‐lighted as green) is added to the C‐terminus (or N‐terminus) to facilitate conjugation.
2. Positive charged residues (K, R, H) are written in blue. Negative charged residues (D, E) are in red.
3. Disorder peptides note: The linear conformation of a disordered peptide is more similar to its natural conformation in the folded protein.

Figure S4 Gene map of the SCMVCpaj405-pET28 (a^+^) expression construct for *E. coli*.

Figure S5 Transformation, ligation, and restriction digestion analysis. An *E. coli* DH5-alpha transformants carrying the synthetic SCMVCpaj405-pUC57-amp (a), and SCMVCpaj405-pET28 (a^+^) gene after shifting (b) using *Nde*1+ *Hind*III; C1, C2, C3, and C4 = colony numbers, and M = 1 kb DNA ladder. SCMVCpaj405 (±948 bp), pUC57-amp (~2710 bp), and pET28 (a^+^) (~5369 bp).

Figure S6 6×His-SCMVCpaj405 recombinantly expressed protein subcellular localization in *E. coli* BL21C+; (a) lanes 1 and 2: total proteins extracted from *E. coli* with SCMVCpaj405-pET28 (a^+^) (induced and uninduced), lane 3: control plasmid only (induced); (b) lanes 1 and 2 = soluble fractions, and pellet; M = unstained protein marker.

## Figures and Tables

**Table 1:** Predicted physiochemical properties of native and fusion SCMVCpaj405 protein genes by ExPASy ProtParam.

S. no.	Protein name	M.wt. (Da)	Sequence length	*p*I	EC1	EC2	Half-life (h)	−R	+R	II	GRAVY	AI
1	Native SCMVCpaj405	33816.62	314	6.80	31525	31400	>10	34	33	40.28	−0.693	53.89
2	6×His-tag SCMVCpaj405	34770.66	321	7.00	31525	31400	>10	34	33	40.00	−0.731	52.71

**Note:** M.wt,. = molecular weight; *p*I = isoelectric point; EC = extinction coefficient at 280 nm (EC1: assuming all Cys residue pairs form cysteine and EC2: assuming all residues of Cys are reduced); −R = number of negative residues; +R = number of positive residues; II = instability index; GRAVY = grand average hydropathy); AI = aliphatic index.

**Table 2:** The ELISA value for the effectiveness and specificity of the raised antiserum in detecting the SCMV in various test samples.

Sample	ELISA reader values	Results
Cell lysate transformed with recombinant plasmid (CYrP)	2.3	+
Cell lysate transformed with plasmid nonrecombinant (CYnP)	0.42	−
Infected sugarcane leaves (ISL)	2.8	+
Healthy sugarcane leaves (HSL)	0.40	−
Purified SCMV rCP (PrCP) as positive control	3.3	+

## Footnotes

1 NCBI (2024). GenBank Overview [online]. Website: https://www.ncbi.nlm.nih.gov/genbank/ [accessed 13 February 2022].

2 ExPASy (2005). ProtParam tool [online]. Website: http://web.expasy.org/protparam [accessed 05 March 2022].

3 I-TASSER (2024). I-TASSER tool [online]. Website: http://zhanglab.ccmb.med.umich.edu/I-TASSER/ [accessed 05 March 2022].

4 SWISS-MODEL (2018). SWISS-MODEL [online]. Website: https://swissmodel.expasy.org [accessed March 2022].

5 Schrödinger, LLC. (2023). PyMOL [online]. Website: https://pymol.org [accessed 29 December 2023].

6 DTU Health Tech. (2024). SignalP 5.0 [online]. Website: https://services.healthtech.dtu.dk/services/SignalP-5.0/ [accessed 23 February 2023].

7 Mitaku Laboratory. (2024). SOSUI series system [online]. Website: https://harrier.nagahama-i-bio.ac.jp/sosui/mobile/ [accessed 24 February 2023].

8 PSORTb Team. (2020). PSORTb version 3.0.3 [online]. Website: https://www.psort.org/psortb/ [25 February 2023].

9 Immune Epitope Database (IEDB). (2024). IEDB [online]. Website: http://www.immuneepitope.org [26 February 2023].

10 GenScript (2023). Optimu Antigen™ design tool [online]. Website: https://www.genscript.com/peptide-antigen-database.html [accessed 26 February 2023].

11 DTU Health Tech. (2024). BepiPred-3.0 [online]. Website: https://services.healthtech.dtu.dk/services/BepiPred-3.0/ [accessed 27 February 2023].
