# Supplementary figures and images for: Development of an immunodiagnostic assay for the detection of Sugarcane mosaic virus
Source: Turk J Biol. 2024 Aug 27;48(6):390–400. doi: 10.55730/1300-0152.2714 (PMC11698196; doi:10.55730/1300-0152.2714)

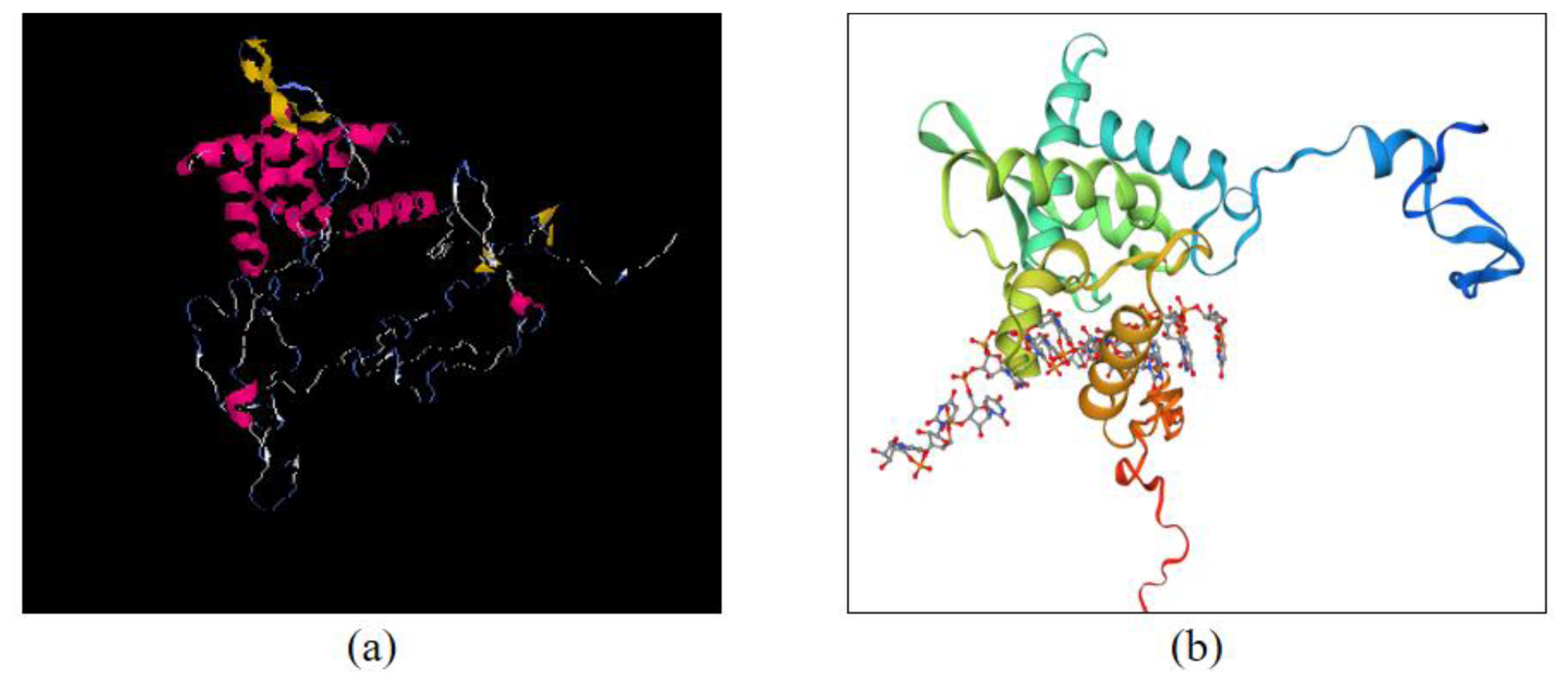

Supplement: Figure S1 — 3D model structures of SCMVCpaj405 protein constructed through I-TASSER (a) and SWISS-MODEL (b). [file tjb-48-06-390s1.tif]

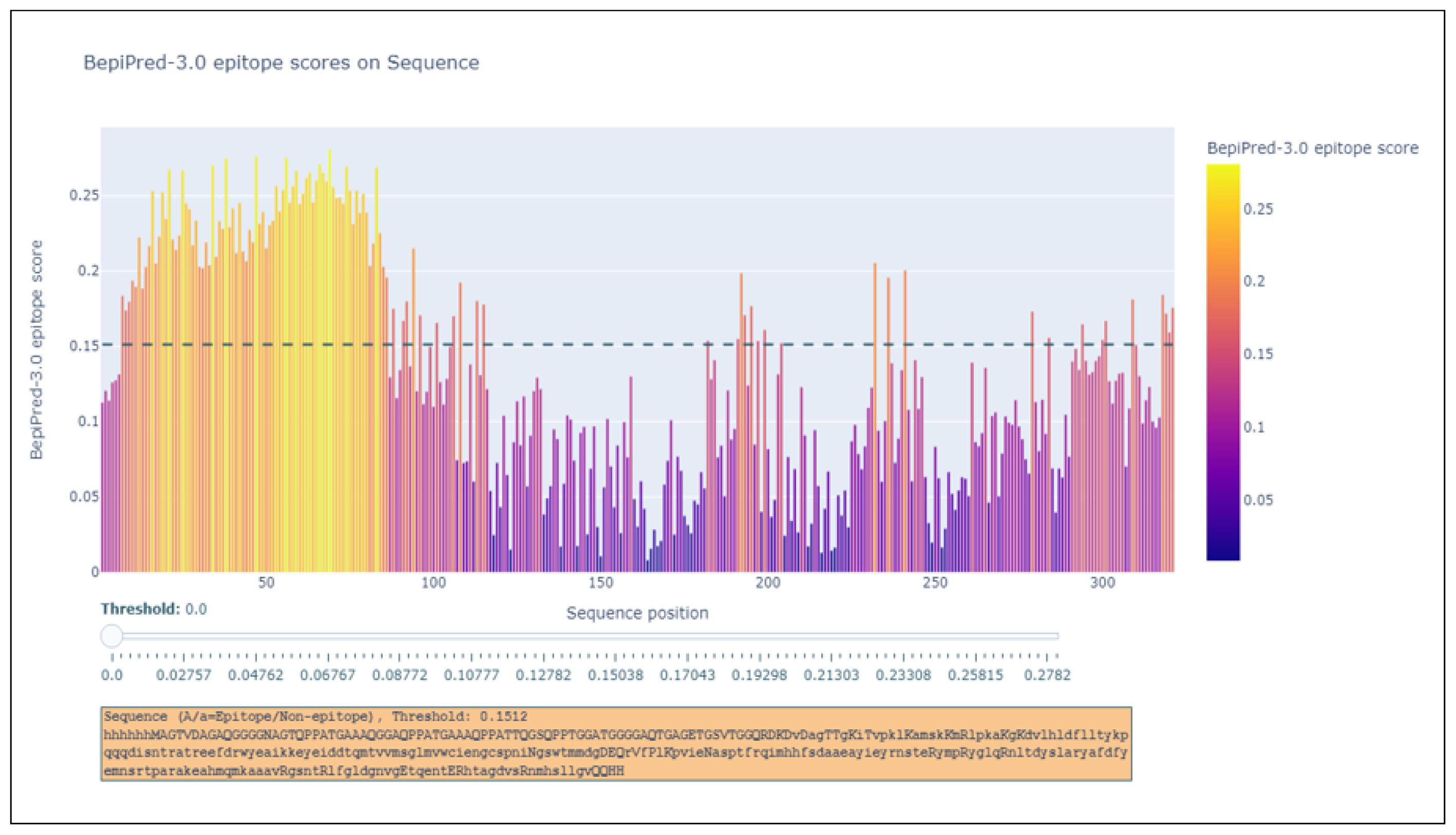

Supplement: Figure S2 — The graphical user interface for BepiPred-3.0 on Cp-SCMV protein. In this interface, the x and y axes are protein sequence positions and BepiPred-3.0 epitope scores. Residues with a higher score are more likely to be part of a B-cell epitope. [file tjb-48-06-390s2.tif]

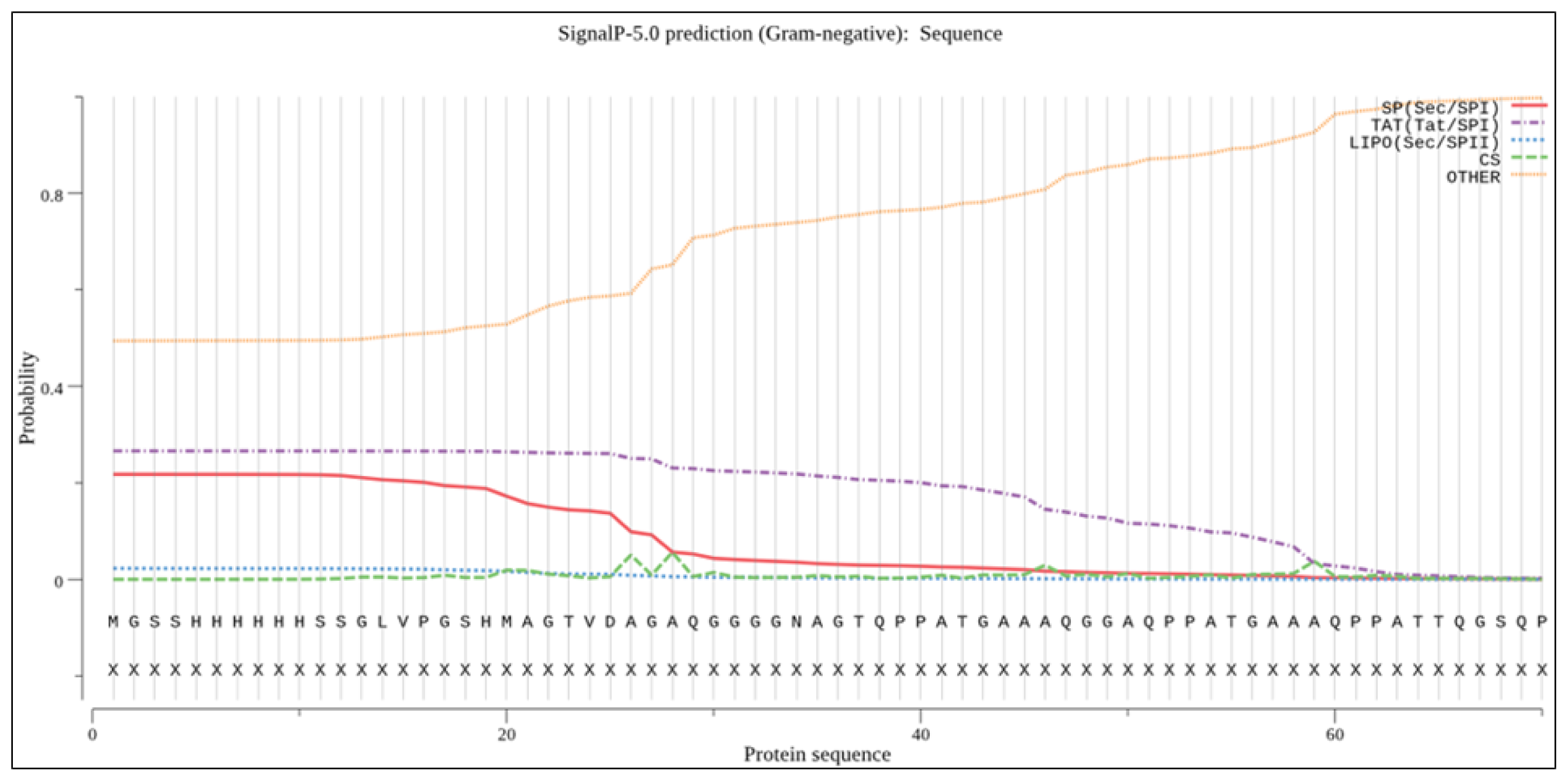

Supplement: Figure S3 — Graphical representation of signal peptide for SCMVCpaj405 protein by Signal-5.0 prediction. [file tjb-48-06-390s3.tif]

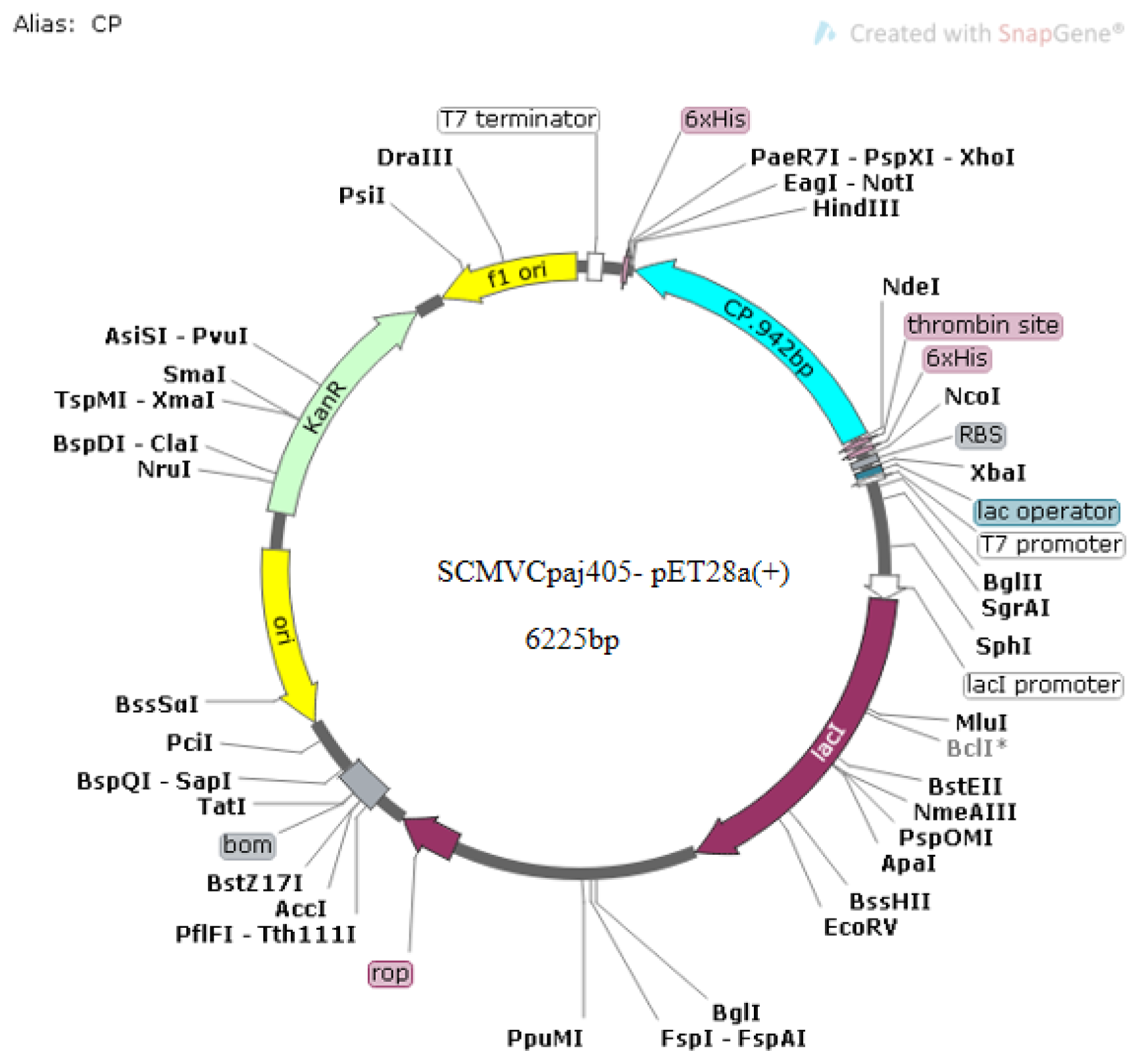

Supplement: Figure S4 — Gene map of the SCMVCpaj405-pET28 (a+) expression construct for E. coli. [file tjb-48-06-390s4.tif]

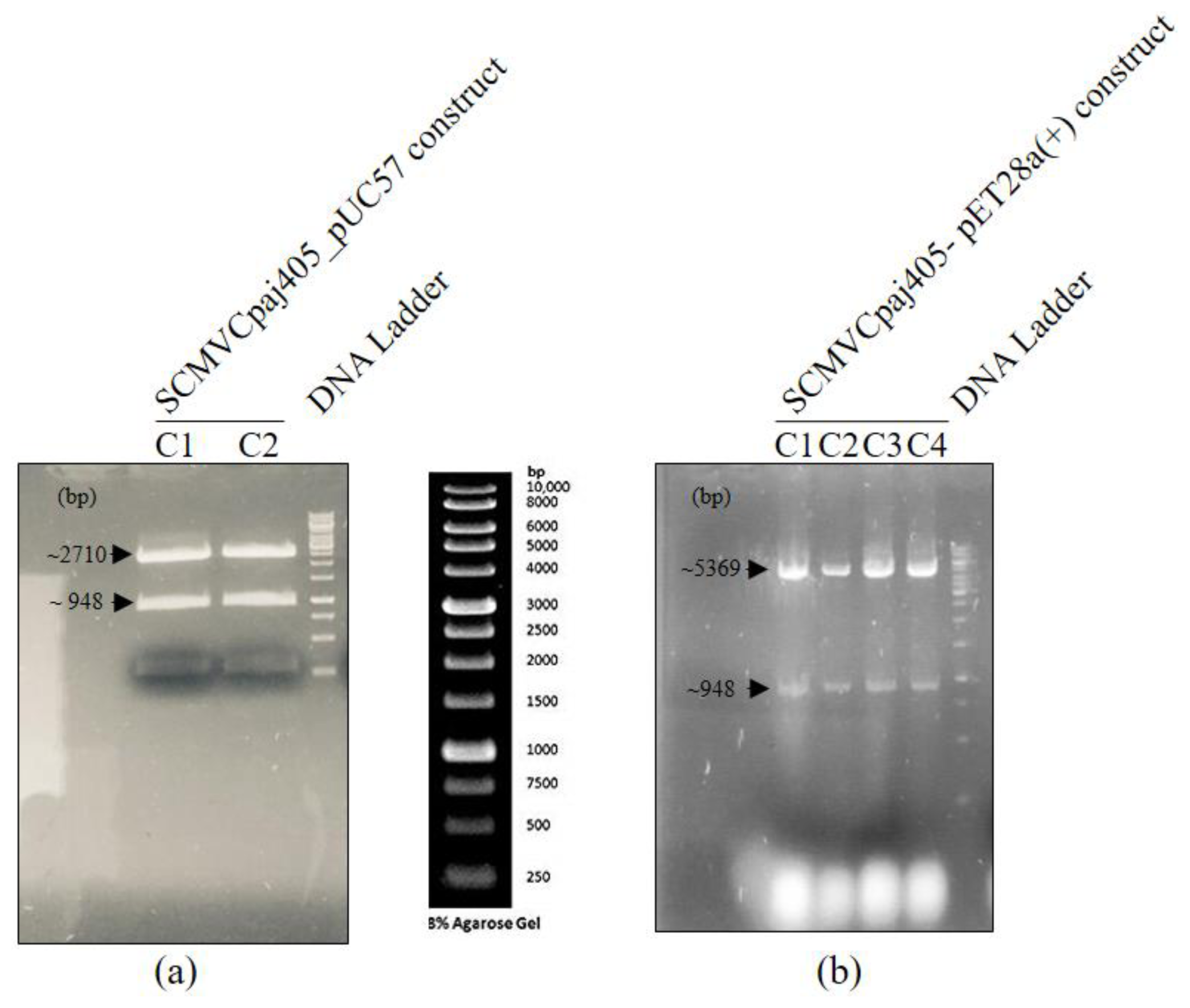

Supplement: Figure S5 — Transformation, ligation, and restriction digestion analysis. An E. coli DH5-alpha transformants carrying the synthetic SCMVCpaj405-pUC57-amp (a), and SCMVCpaj405-pET28 (a+) gene after shifting (b) using Nde1+ HindIII; C1, C2, C3, and C4 = colony numbers, and M = 1 kb DNA ladder. SCMVCpaj405 (±948 bp), pUC57-amp (~2710 bp), and pET28 (a+) (~5369 bp). [file tjb-48-06-390s5.tif]

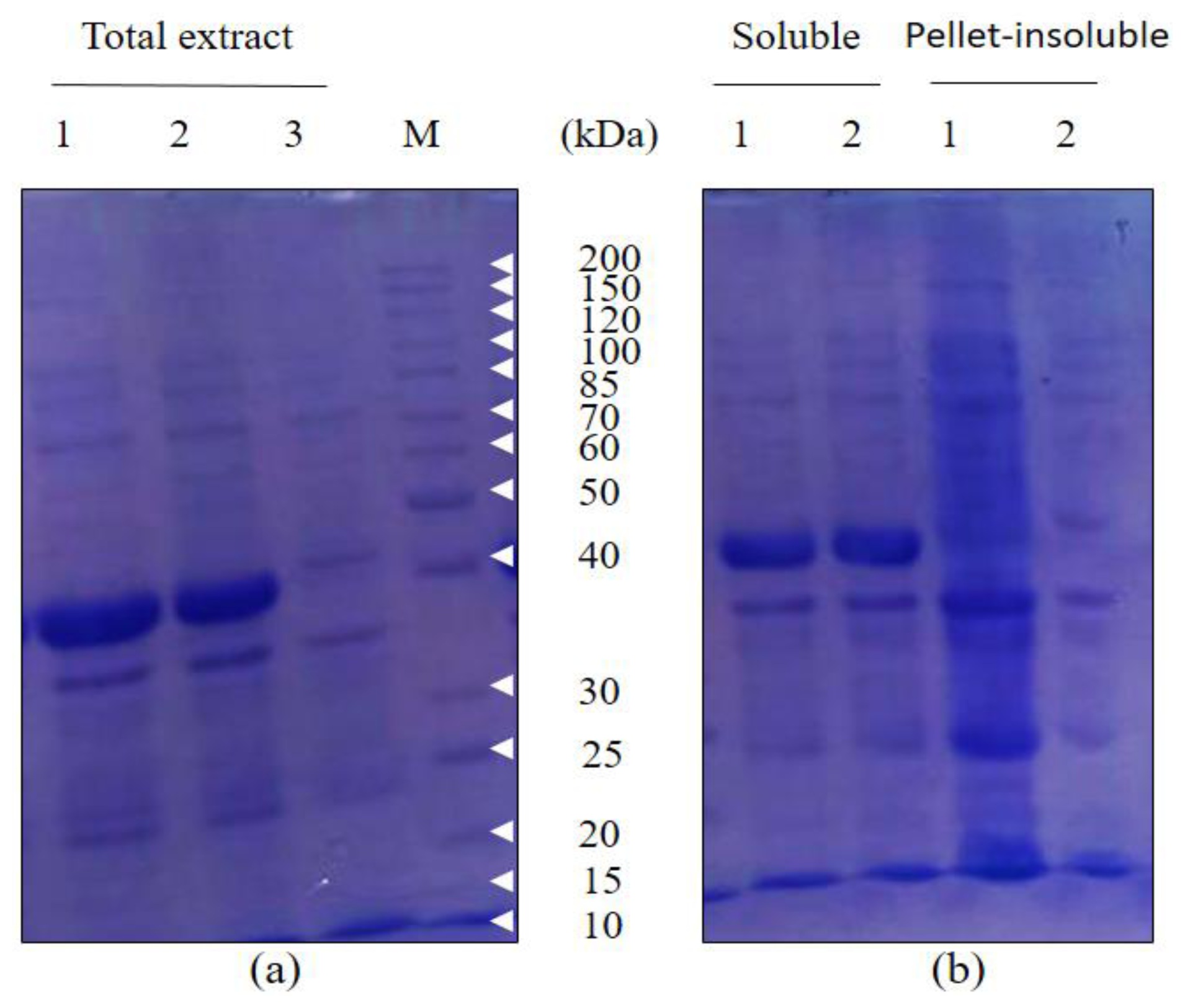

Supplement: Figure S6 — 6×His-SCMVCpaj405 recombinantly expressed protein subcellular localization in E. coli BL21C+; (a) lanes 1 and 2: total proteins extracted from E. coli with SCMVCpaj405-pET28 (a+) (induced and uninduced), lane 3: control plasmid only (induced); (b) lanes 1 and 2 = soluble fractions, and pellet; M = unstained protein marker. [file tjb-48-06-390s6.tif]
